# Transperineal laser ablation for 300-gram benign prostatic hyperplasia in an inoperable high-risk patient: Expanding the therapeutic horizon

**DOI:** 10.1016/j.eucr.2026.103591

**Published:** 2026-08-26

**Authors:** A.A. Abou Taleb

**Affiliations:** a Urology, Benha University, Egypt; b UroPro Centers, Egypt

**Keywords:** Benign prostatic hyperplasia, Transperineal laser ablation, Minimally invasive therapy, Urinary retention

## Abstract

Transperineal laser ablation (TPLA) is an emerging minimally invasive option for benign prostatic hyperplasia, but its role in massive glands remains undefined. We report a 78-year-old catheter-dependent man with a 300-g prostate, severe cardiopulmonary comorbidity, left ventricular ejection fraction of 25%, and mandatory dual antiplatelet therapy, making standard surgery unsafe. Total-gland TPLA was performed under local anesthesia using eight fibers and 42,300 J without complications. The patient became catheter-free after 15 days, with Qmax improving to 19.3 mL/s and prostate volume decreasing to 90 g at six months. TPLA may be feasible in selected high-risk patients with extreme BPH.

## Highlights

• First reported total-gland TPLA for a 300-g prostate.
• TPLA was performed under local anesthesia in a prohibitive-risk patient.
• Eight-fiber ablation delivered 42,300 J without perioperative complications.
• Catheter-free spontaneous voiding returned 15 days after TPLA.
• Qmax improved to 19.3 mL/s with PVR 0 mL at 6 months.

## 1 Introduction

Benign prostatic hyperplasia (BPH) is a prevalent urological condition, with histological evidence in up to 80% of men aged 80 years and older. BPH often leads to bladder outlet obstruction and lower urinary tract symptoms (LUTS), including hesitancy, weak stream, nocturia, and, in advanced cases, acute urinary retention and upper tract deterioration.^1^ Medical therapy, including α-blockers and 5α-reductase inhibitors, is first-line, but many patients fail to achieve satisfactory control or progress to surgery.^2^

For decades, transurethral resection of the prostate (TURP) has been considered the gold standard surgical treatment for moderate to large prostate glands.^3^ In massive BPH—typically exceeding 80–100 grams—open simple prostatectomy or laser enucleation is often required, but these interventions carry substantial risk and are frequently contraindicated in patients with significant cardiopulmonary comorbidities or ongoing anticoagulation, leaving such high-risk patients with an urgent need for less invasive, organ-preserving techniques.^4^

Over the last decade, several minimally invasive therapies have emerged to bridge this gap, including prostatic artery embolization, water vapor thermal therapy, and prostatic urethral lift.^5^ Among these, transperineal laser ablation (TPLA) has recently gained attention as a promising non-resective modality for the treatment of symptomatic BPH. TPLA utilizes fine-caliber optical fibers to deliver precise, high-energy diode laser-induced coagulative necrosis to the adenomatous tissue through a percutaneous transperineal approach. Unlike transurethral techniques, TPLA spares the urethral channel and preserves ejaculation, and it can be performed under local anesthesia, making it particularly attractive for fragile or compromised patients.^6^

However, TPLA use has remained limited to glands under 100 g; no reports have described its use in huge prostates, and its feasibility in massive BPH remains unexplored.

At our center, we have performed more than 210 TPLA procedures for symptomatic BPH over the past three years, the largest gland previously treated being 170 g. The current patient presented with a 300-g gland and severe cardiopulmonary comorbidities that precluded any standard approach. Given the absence of published data and of alternative therapeutic options, we considered TPLA an individualized solution; he was extensively counselled regarding its novel application and uncertain outcomes in glands of this size, and provided written informed consent.

This case highlights the procedural feasibility, safety, and promising early functional outcomes of TPLA in a patient “beyond surgical limits,” and expands the potential therapeutic horizon for minimally invasive management of extreme BPH.

## 2 Case presentation

A 78-year-old man was referred to our urology center after nine months of continuous catheterization due to complete urinary retention. Beforehand, he had worsening symptoms — weak stream, hesitancy, incomplete emptying, and nocturia — until he lost the ability to void entirely. Several trials of catheter removal were unsuccessful.

His overall health posed a significant challenge. He had COPD with noticeable shortness of breath on exertion, and a seriously weakened heart — his left ventricular ejection fraction was just 25%. A year earlier, he had undergone coronary stenting, and his cardiologist had made it clear that his dual antiplatelet therapy, including clopidogrel, could not be stopped or bridged under any circumstances given his high clotting risk. Because of this, multiple urology centers had turned him away, including for procedures like HoLEP, deeming him too high-risk for intervention despite his massively enlarged prostate.

On examination, he was stable — blood pressure 130/80 mmHg, heart rate 76 bpm, and oxygen saturation 95% on room air. His abdomen was soft and non-tender, and digital rectal examination revealed a significantly enlarged, firm prostate with an obliterated median sulcus and prominent central protrusion, though no suspicious nodules were felt. The catheter was draining clear urine with no signs of infection or bleeding.

**Fig. 1:**
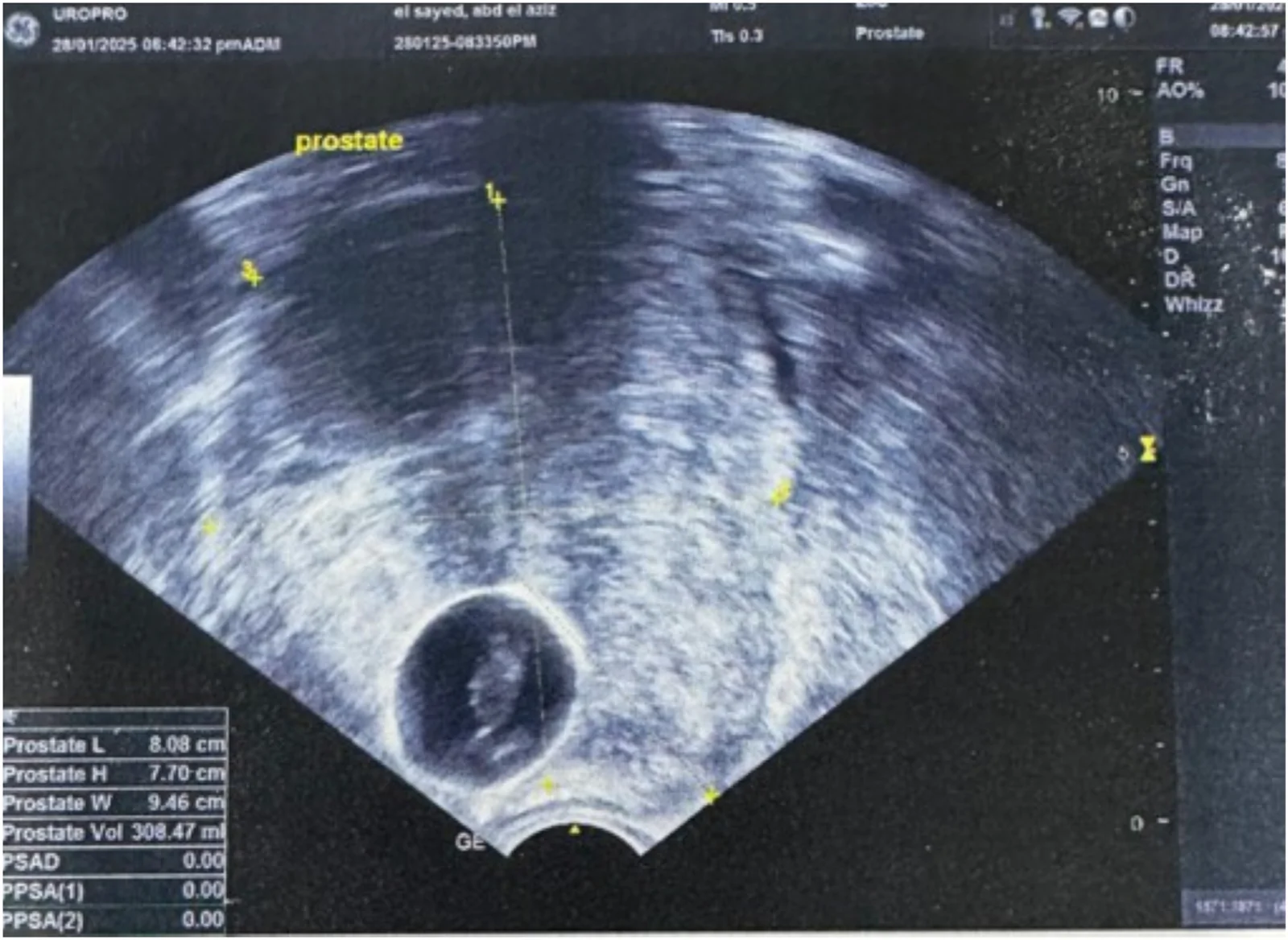
Sonography of the patient demonstrating Giant Benign Prostatic Hyperplasia (BPH) with a calculated volume of 308 mL.

Lab work showed stable kidney function, and his PSA was 3.6 ng/mL. Transrectal ultrasound confirmed a markedly enlarged prostate estimated at 312 g — so large that it couldn't be measured in a single imaging plane. The median lobe alone measured 165 g, with lateral lobe extension of 47 g, though the prostatic capsule remained intact. No suspicious lesions were identified. Uroflowmetry was not possible given the indwelling catheter. Fig. 1.

Given the combination of massive BPH (∼300 g), prolonged catheter dependence, and extremely high anesthetic and bleeding risk, conventional surgery was simply not a safe option. We therefore offered the patient transperineal laser ablation (TPLA) — a novel, minimally invasive alternative. It was the first time our center had attempted TPLA in a prostate of this size, and its effectiveness at such extreme volumes was unknown.

The procedure was performed in an interventional suite under local anesthesia alone — no general or spinal anesthesia was needed. It was carried out in a single session divided into two stages: the first targeted the central median lobe using four laser fibers, and the second addressed the right and left lateral lobes with four additional fibers — eight fibers in total to ablate the entire gland.

**Fig. 2:**
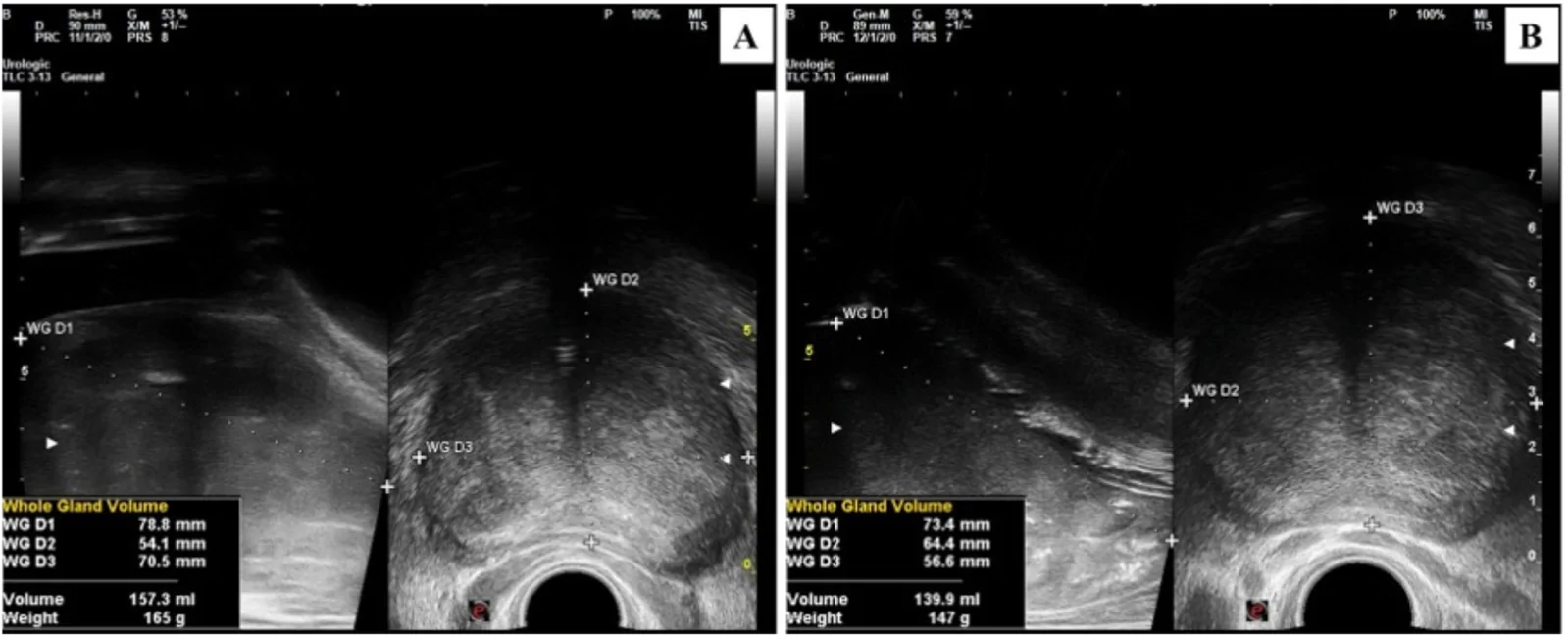
TRUS assessment of prostate. A: Median lobe; B: Lateral lobe.

Planning began with high-resolution transrectal ultrasound to map the prostate and its relationship to surrounding structures, including the urethra, bladder neck, apex, and capsule (Fig. 2). Eight transperineal access points were chosen to ensure optimal distribution of thermal energy across the enlarged gland. Each entry site was numbed with local lidocaine, and bilateral periprostatic nerve blocks were given for additional pain control.

The first phase targeted the central adenoma (∼165 g). Four optical fibers were inserted through 21-gauge Chiba needles under real-time ultrasound guidance, positioned within the core tissue while keeping at least 8–10 mm from the urethra and capsule. A 22 Fr three-way Foley catheter was placed, and the bladder was filled with ∼300 cc of saline to push the bladder wall away from the ablation zone and reduce thermal injury risk. Continuous saline irrigation was also maintained throughout for cooling.

Laser energy was delivered via the Echolaser system (Elesta) using a two-step pullback technique per fiber to ensure thorough central coverage. Once the central zone was adequately ablated, four additional fibers — two per side — were directed at the lateral lobes using the same careful margins and continuous ultrasound monitoring. In total, 42,300 J were delivered evenly across all eight fibers at 5400 J each, producing symmetrical thermal lesions while preserving surrounding structures. The procedure was completed in 58 minutes without any complications.

Of note, prostate volumes measured at the initial outpatient visit and again intraoperatively differed by less than 5%, despite being performed by different operators on different machines — a variation considered clinically insignificant.

After the procedure, the catheter was kept in place for 15 days to support drainage and allow the tissue to recover from the expected post-ablation swelling. The patient was discharged on a five-day course of IV ceftriaxone, along with oral prednisolone and meloxicam to control inflammation and discomfort. He arrived home in stable condition with follow-up arranged for uroflowmetry and imaging.

At the 15-day follow-up visit, the catheter was removed without incident. Uroflowmetry performed at that time showed a maximum flow rate (Q_max_) of 7.6 mL/sec with a voided volume of 190 mL and a post-void residual (PVR) of 70 mL. The patient reported the return of spontaneous voiding for the first time in nearly one year and expressed satisfaction with his progress. There were no reports of fever, hematuria, or urinary tract infection. PSA decreased to 2.2 ng/mL following the procedure.

**Fig. 3:**
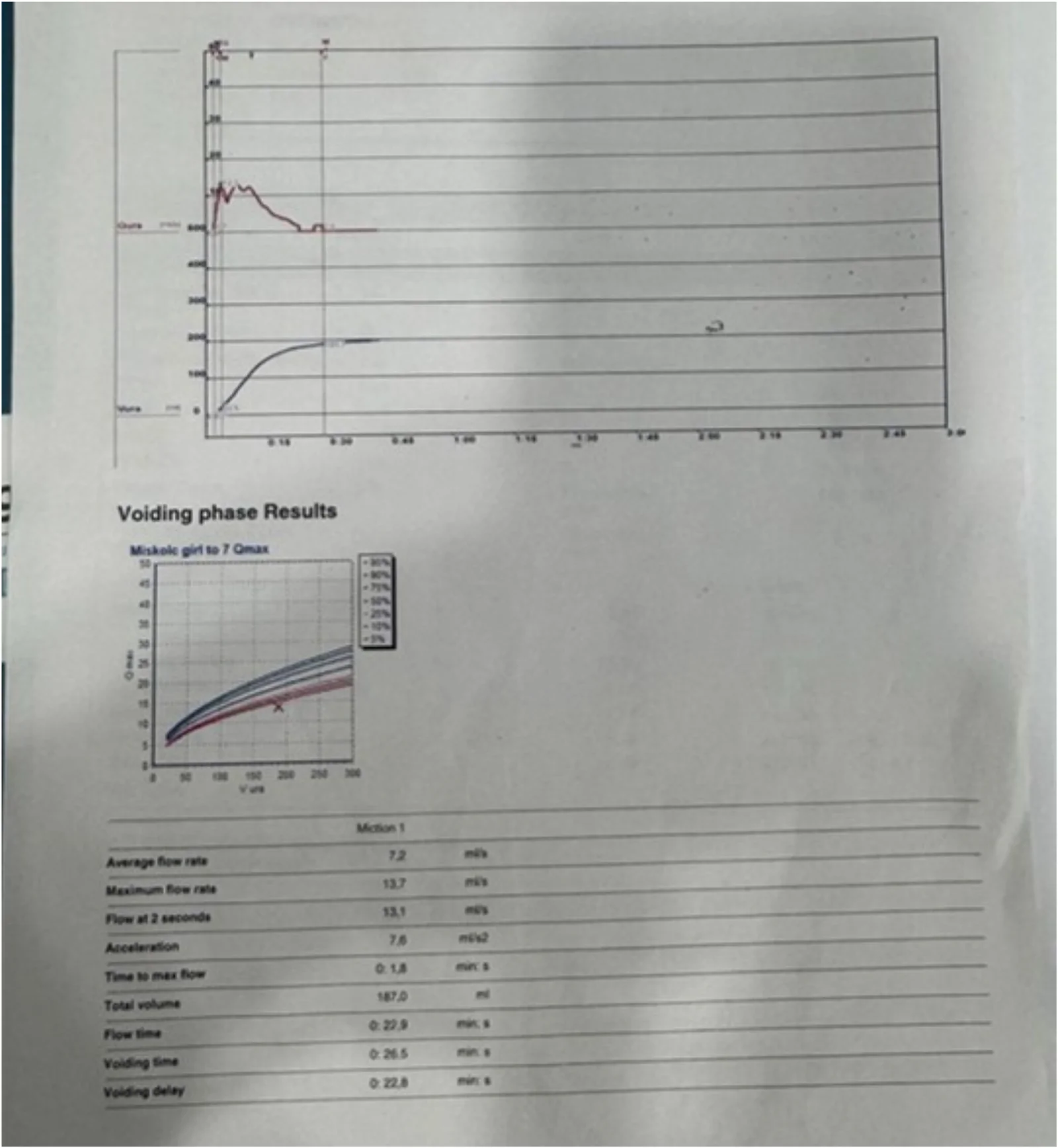
Uroflowmetry tracing showing improved flow and complete bladder emptying at three months post-TPLA.

At three months post-procedure, the patient continued to void comfortably and remained catheter-free. Uroflowmetry demonstrated a Q_max_ of 13.7 mL/sec and complete bladder emptying, with no post-void residual (PVR = 0 mL) (Fig. 3). Transrectal ultrasound at that time showed a substantial reduction in prostate size, with a final measured volume of approximately 90 g. These findings were further confirmed on follow-up MRI. The patient reported a complete resolution of his prior urinary symptoms and was able to resume daily activities without the need for further urological intervention or medical therapy.

**Fig. 4:**
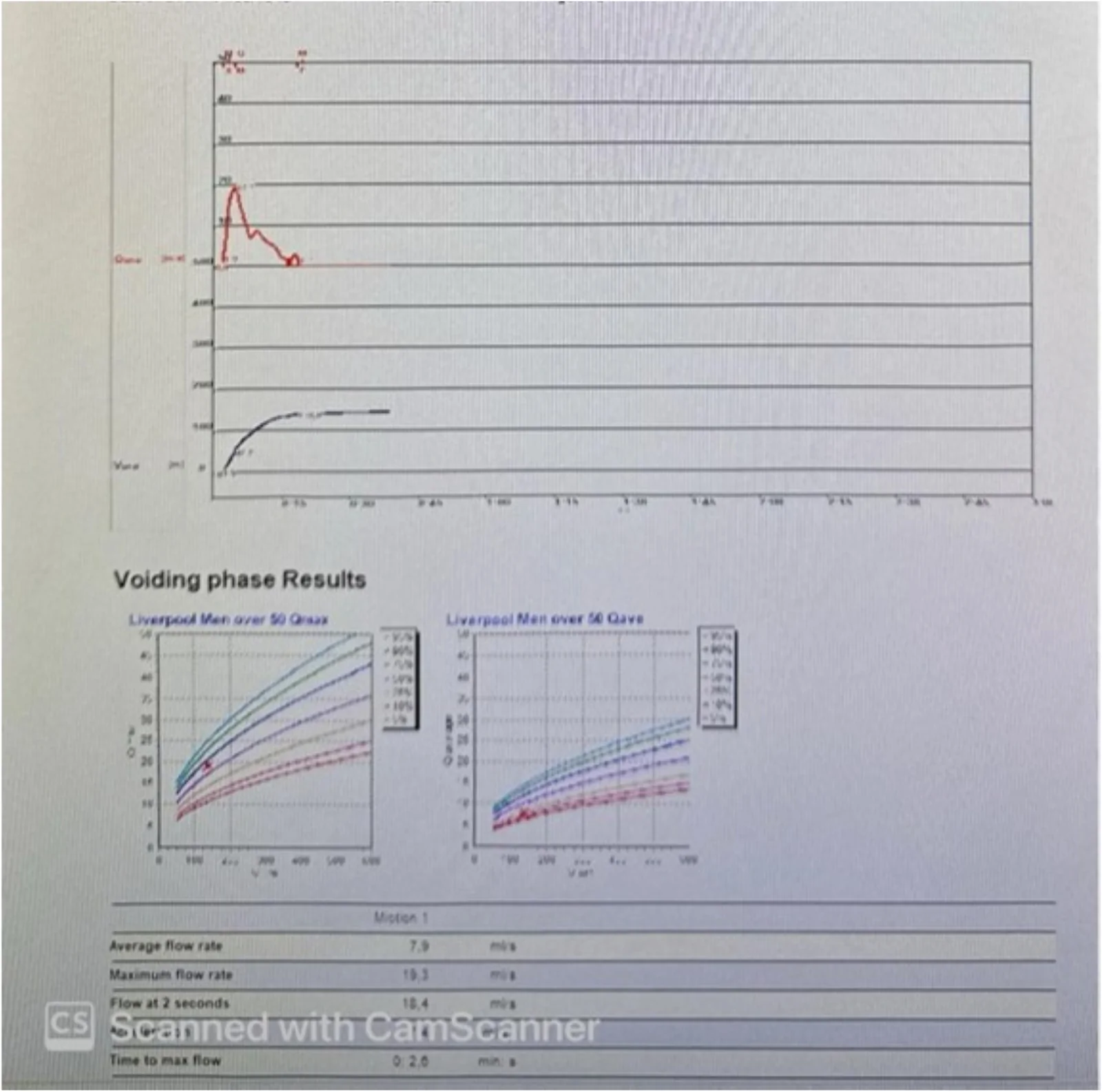
Uroflowmetry at six months post-TPLA.

At six months post-procedure, the patient reported further improvement with better urinary flow and greater comfort. Uroflowmetry showed a Q_max_ of 19.3 mL/s with no post-void residual urine (PVR = 0 mL) (Fig. 4). Transrectal ultrasound demonstrated a stable prostate size of approximately 90 g.

## 3 Discussion

TPLA, a novel percutaneous laser-based technique, has gained traction as a minimally invasive alternative to TURP, HoLEP and open prostatectomy in selected patients with moderate prostate volumes. The technique's distinguishing features—transperineal access, real-time ultrasound guidance, urethral sparing, and feasibility under local anesthesia—render it uniquely advantageous in high-risk candidates.^7^ In the present case, the patient's severe cardiopulmonary status (LVEF 25%) and chronic antiplatelet use rendered him unsuitable for general, spinal, or even prolonged sedation. Thus, TPLA offered a viable intervention where no other option was safely available.

In a multi-center retrospective study by Pacella et al.,^8^ TPLA significantly improved IPSS, Qmax, PVR, and QoL at 6 and 12 months in prostates averaging 75–88 mL, with complication rates under 5% and virtually no need for transfusion or general anesthesia; catheter-dependent patients, such as ours, were not excluded. Our case extends this beyond previously reported thresholds and is, to our knowledge, the first report of TPLA in a 300-g prostate. A single session with eight fibers and over 42,000 J—well above standard protocols—was completed without intraoperative or postoperative complications on an outpatient basis, and the patient achieved spontaneous voiding within 15 days, with progressive improvement in Qmax and resolution of PVR by three months.

Current pooled data, as reviewed by Tafuri et al.,^9^ confirm that TPLA consistently improves Q_max_ (from 8.7 to over 14 mL/s), reduces PVR (from ∼90 to <30 mL), and lowers IPSS scores (by over 60%) within 6 months. Importantly, sexual function is preserved or even improved in selected cohorts, likely due to bladder neck and ejaculatory pathway preservation—an advantage over TURP and laser enucleation techniques, which carry retrograde ejaculation rates of 60–80%. Although our patient was not sexually active, the urethral-sparing nature of the procedure was instrumental in preventing postoperative irritative symptoms and preserving continence.

This case also demonstrates that TPLA is technically feasible in massively enlarged glands. Multiple fiber tracts, with careful respect for the urethra, capsule, and bladder neck, maintained thermal symmetry without adverse effects. Prostate volume fell from 312 to 90 g within three months, suggesting that even extremely large glands can be effectively debulked without endoscopic surgery, open operation, or general anesthesia.

Long-term data are still needed: no randomized trials have compared TPLA with standard treatments in prostates over 100 g, and durability beyond 12 months remains unclear. Nonetheless, its safety profile, minimal anesthesia requirements, and outpatient feasibility make TPLA a compelling option for patients too high-risk for conventional surgery.

Prostatic artery embolization (PAE) is another minimally invasive option for poor surgical candidates, particularly those with significant comorbidities or high bleeding risk. Compared with TPLA, PAE is performed through an endovascular approach and can treat very large prostate volumes without direct access to the gland.^10^ However, PAE requires arterial catheterization, contrast exposure, and successful identification and embolization of prostatic arterial branches, which may be difficult in patients with extensive vascular disease, prior pelvic interventions, or unfavorable anatomy. Response after PAE may also be variable, with symptom improvement often gradual over several months.^11^ In contrast, TPLA provides direct, targeted ablation of adenomatous tissue under real-time ultrasound guidance, while avoiding vascular manipulation and allowing treatment under local anesthesia alone.^7^

Therefore, PAE remains an important alternative, particularly when endovascular expertise is available or when gland size and anatomy are unfavorable for transperineal access, whereas TPLA may be particularly attractive in selected high-risk patients requiring precise tissue reduction with minimal physiological stress. Future comparative studies are needed to define the optimal role of each technique in patients with extreme prostate enlargement.

## 4 Conclusion

TPLA may represent a paradigm shift in the management of extreme BPH in high-risk individuals. Our report documents the first successful total-gland TPLA in a 300-g prostate under local anesthesia, with favourable functional and anatomical outcomes and no complications. This case expands the therapeutic horizon of TPLA and supports its consideration as a viable option for patients previously deemed beyond surgical reach.

## Ethics declaration

Written informed consent to take part in the study and to publish the article has been obtained from all participants or their legal representatives. The privacy rights of participants have been observed.

This study was performed in compliance with relevant laws, regulatory frameworks and guidelines where the research took place. This study was approved by the Benha University, Faculty of Medicine. (Approval No. RC.6.3.2026)

## References

[1] Lim K.B. (Jul 2017). Epidemiology of clinical benign prostatic hyperplasia. Asian J Urol.

[2] Macey M.R., Raynor M.C. (Sep 2016). Medical and surgical treatment modalities for lower urinary tract symptoms in the Male patient secondary to benign prostatic hyperplasia: a review. Semin Interv Radiol.

[3] Porto J.G., Bhatia A.M., Bhat A. (2024/11/15 2024). Evaluating transurethral resection of the prostate over twenty years: a systematic review and meta-analysis of randomized clinical trials. World J Urol.

[4] Checcucci E., Veccia A., De Cillis S. (Nov 2021). New ultra-minimally invasive surgical treatment for benign prostatic hyperplasia: a systematic review and analysis of comparative outcomes. Eur Urol Open Sci.

[5] Bilhim T., Betschart P., Lyatoshinsky P., Müllhaupt G., Abt D. (Apr 2022). Minimally invasive therapies for benign prostatic obstruction: a review of currently available techniques including prostatic artery embolization, water vapor thermal therapy, prostatic urethral lift, temporary implantable nitinol device and aquablation. Cardiovasc Interv Radiol.

[6] Tzelves L., Nagasubramanian S., Pinitas A. (Jan-Dec 2023). Transperineal laser ablation as a new minimally invasive surgical therapy for benign prostatic hyperplasia: a systematic review of existing literature. Ther Adv Urol.

[7] Lo Re M., Polverino P., Rivetti A. (Jul 10 2024). Transperineal laser ablation (TPLA) of the prostate for benign prostatic obstruction: the first 100 patients cohort of a prospective, single-center study. World J Urol.

[8] Pacella C.M., Patelli G., Iapicca G. (Jun 2020). Transperineal laser ablation for percutaneous treatment of benign prostatic hyperplasia: a feasibility study. Results at 6 and 12 months from a retrospective multi-centric study. Prostate Cancer Prostatic Dis.

[9] Tafuri A., Panunzio A., De Carlo F. (Feb 26 2023). Transperineal laser ablation for benign prostatic enlargement: a systematic review and pooled analysis of pilot studies. J Clin Med.

[10] Altman R., Ferreira R., Barragan C. (Jan 28 2024). Comparing prostatic artery embolization to surgical and minimally invasive procedures for the treatment of benign prostatic hyperplasia: a systematic review and meta-analysis. BMC Urol.

[11] Dias U.S., de Moura M.R.L., Viana P.C.C. (Sep-Oct 2021). Prostatic artery embolization: indications, preparation, techniques, imaging evaluation, reporting, and complications. Radiographics.

